# ‘Walking in the shoes of our patients’: a scoping review of healthcare professionals learning from the simulation of patient illness experiences

**DOI:** 10.1186/s41077-021-00194-w

**Published:** 2021-12-04

**Authors:** Milda Karvelytė, Janet Rogers, Gerard J. Gormley

**Affiliations:** 1 Bachelor of Science in Human Biology, Queen’s University Belfast, Belfast, UK; 2 Centre for Medical Education, Queen’s University Belfast, Belfast, UK

**Keywords:** Simulation-based education, Health profession, Illness experiences

## Abstract

**Background:**

Health professionals who have experienced ill-health appear to demonstrate greater empathy towards their patients. Simulation can afford learners opportunities to experience aspects of illness, but to date, there has been no overarching review of the extent of this practice or the impact on empathic skills.

**Objective:**

To determine from the evidence—what is known about simulation-based learning methods of creating illness experiences for health professions and the impact on their empathic skills.

**Study selection:**

Arksey and O’Malley’s methodological framework informed our scoping review of articles relevant to our research question. Three databases (MEDLINE, Embase and Web of Science) were searched, and a sample of 516 citations was screened. Following review and application of our exclusion criteria, 77 articles were selected to be included in this review.

**Findings:**

Of the 77 articles, 52 (68%) originated from the USA, 37 (48%) of studies were qualitative based and 17 (22%) used a mixed-methods model. Of all the articles in our scope, the majority (87%) reported a positive impact and range of emotions evoked on learners. However, some studies observed more negative effects and additional debriefing was required post-simulation. Learners were noted to internalise perceived experiences of illness and to critically reflect on their empathic role as healthcare providers.

**Conclusions:**

A diverse range of simulation methods and techniques, evoking an emotional and embodied experience, appear to have a positive impact on empathy and could be argued as offering a complementary approach in healthcare education; however, the long-term impact remains largely unknown.

**Supplementary Information:**

The online version contains supplementary material available at 10.1186/s41077-021-00194-w.

## Introduction

Healthcare is a humane discipline grounded in compassion [1–15]. Healthcare professionals (HCPs) are obliged to provide empathic care, leading to many benefits for patients. In addition to patient satisfaction, empathetic care is associated with greater diagnostic accuracy, compliance with treatment and decreased rates of clinical errors [2–4, 6–12]. For example, doctors who provide empathetic care towards their patients are less likely to receive complaints and medical negligence claims [1, 16]. Moreover, providing empathetic care can enhance HCPs’ own satisfaction and wellbeing [17, 18].

### The empathy challenge

Empathy is considered to be the ability to sense others’ emotions and understand what they may be feeling. By nature, empathy is considered a complex multidimensional and interpersonal state [1, 19, 20]. Empathy is influenced by many factors, including context, therefore making it challenging to measure consistently [1, 19, 20]. Nevertheless, the ability to understand a patient’s emotional state from their point of view is foundational to humanistic healthcare [1, 19, 21–25]. However, evidence would suggest there are challenges in providing the best of empathic healthcare for patients. In addition to the huge pressures in modern healthcare environments, research would suggest that HCPs’ level of empathy is at risk of declining during their training [21, 26–29].

An array of educational strategies has been established to promote HCPs’ empathy towards their patients. However, there often can be a disconnect between what is *learnt* about empathy and what is *demonstrated* in clinical practice. For example, in terms of intellectual learning about empathy, increasing students’ empathy through discussing and reading literature might contribute positively in the short term, though results have not been consistent. Importantly, we understand that experiential forms of learning can offer learners a deeper understanding of *self*—compared to more intellectual modes of learning.

### Experiential learning of empathy: *stepping in the shoes of patients?*

Experiencing ill-health can be a powerful motivator for HCPs in developing empathy towards patients, not only gaining a greater appreciation of their illness lifeworld (i.e. the immediate experiences, activities and contacts that make up the world of an individual) but also demonstrating empathic care [30, 31]. Experiencing illness can provide a radical shift in perception ‘from the normal vertical to the dreaded horizontal’ [30]*.* For example, through an interpretative phenomenological lens, Fox et al. explored the lived experiences of GPs who themselves have had ill-health [31]. They concluded that experiencing illness promoted a more holistic approach towards patients; in so doing brought a greater understanding about their intellectual and emotional responses in empathic care [31]. While we would not want learners to experience actual ill-health, there have been attempts to harness simulation to recreate illness experiences (i.e. the features experienced by a person who lives with a particular condition) for learners to gain a deeper insight of illness experiences—in the hope of improving their future empathic care towards patients.

### Simulation and recreating illness experiences

Simulation is an established teaching approach in HCP education. Guided by pedagogy, simulation-based education (SBE) aims to create important learning opportunities that may not be readily available or suitable in clinical environments [32–41]. Whether refining craft skills such as laparoscopy or rehearsing how to manage high acuity/time-dependent events such as cardiac arrest—SBE aims to advance practitioners’ skills in a safe and guided fashion. The creation of such realities in SBE is often focused on enabling health professionals to ‘step into the shoes’ of their future *professional-self* (i.e. extending them beyond limitations of individual capacities in a supportive and scaffolded approach). However, we are aware of a number of studies that redirect the focus to *being a patient*; in essence, harnessing simulation to allow HCPs to have an experiential (i.e. involving or based on experience and observation) experience of illness, encouraging critical reflection in their professional development and ultimately aiming to cultivate greater empathic care towards patients [42–44]. Simulating the features of illness experiences, in theory, could provide learners with insights of patients’ experiences and gain a more holistic sense of illness [35, 45–48].

The aim of this study was to determine what is known about SBE methods of creating illness and care experiences for HCPs and the impact on their empathic skills.

## Methods

### Methodological approach

We choose a scoping review methodology to *map* the literature on what is known about SBE methods of creating illness experiences for HCPs and the impact on their empathic skills. This methodological approach aligned with our research aim and our subjectivist orientation, drawing upon a wide range of knowledge derived from various epistemological positions in order to provide a rich understanding of the phenomenon under investigation (i.e. to be inclusive of studies that subjectively and objectively investigate the holistic experience of simulating illness).

### Research team

The research team comprised of individuals from different backgrounds—including an undergraduate student (MK), an Academic General Practitioner (JR) and a Clinical Professor of Simulation (GJG). The research team met continually and engaged reflexively with each other throughout the study. Some members of the research team live with illness.

### Scoping literature review method

Arksey and O’Malley’s methodological framework guided our scoping review of the literature performing the first five stages (stage 1: identifying the research question; stage 2: identifying relevant articles; stage 3: article selection; stage 4: charting the data; and finally stage 5: collating, summarising and reporting the results) [49]*.* In keeping with this framework, our aim was to map the *contours* of the evidence base, including the gaps, rather than formally appraising the quality of evidence [49, 50].

#### Stage 1: Identifying the research question

Guided by our research aim, we applied the ‘Population, Situation’ tool to develop our research question [51]. We determined the *population* to be HCPs (and students) and simulation-based learning methods of creating illness experiences to be the *situation*. Specifically, our scoping review set out to address the following research objectives:

1. Identify methods and details of creating simulated illness and treatment experiences
2. Consider the impact, if any, on cultivating HCPs’ empathic skills

#### Stage 2: Identifying relevant articles

Aligned to our research question, we developed a search strategy in consultation with a librarian who had expertise in health-related databases (Table 1). In November 2020, three databases were searched: MEDLINE, Embase and Web of Science using our full search strategy and terms.

**Table 1:** Search terms used for database searches

Simulation type/equipment	Perceived emotion	Condition/symptom
Experien* learning*	Empath*	Diabet*
Simulat* training*	Emotional* intelligence*	Old* age*
Illness* simulat*	Patienthood*	Auditor* hallucination*
Disease* simulat*	Emotional* capacit*	Melanoma*
Deaf* simulat*	Compassion*	Deaf*
Mental* illness* simulat*	Emotional* abilit*	COPD*
Dementia* simulat*	Empath* scale*	Chronic* pain*
Sim-spec*	Attentive* listen*	Glaucoma*
Embod* experien*	Bedside* manner*	Macular* degenerat*
Aging* game*	Share* humanit*	Aging*
Embod*	Doctor* understand*	IBD (inflammatory bowel disease*)
Immers* learn*	Patient* experienc*	Mental* illness*
Diabet* mellitus* simulat*	Patient* perspective*	Mental* disorder*
Learn* by living*	Patient* concern*	ABI (acute brain injur*)
Patient* simulat*	Patient* feeling*	Urinary incontinenc*
Simulat* patient*	Physician* understand*	Restrictive pulmonary disease*
Virtual* simulat*		Alzheimer's disease*
Simulat* experien*		Dementia*
Deafness* simulat*		Visual* impair*
Hallucination* simulat*		Diabet* mellitus*
Auditor* hallucination* simulat*		Hallucination*
Schizophrenia* simulat*		Schizophrenia*
Melanoma* simulat*		Obes*
Transfer* tattoo*		Ostom*
Temporar* tattoo*		Wheelchair*
Melanoma* tattoo*		Stom*
Simulat* software*		
Arthriti* simulat*		
Arthriti* glove*		
Pain* simulat*		
Back* pain* simulat*		
Paralysis* simulat*		
Stroke* simulat*		
Disabilit* simulat*		
Hear* loss* simulat*		
Hear* impair* simulat*		
Blind* simulat*		
Vision* loss* simulat*		
Blindness* simulat*		
Retinal* detach* simulat*		
Cataract* simulat*		
Macular* degeneration* simulat*		
Simulat* goggle*		
Simulat* goggl*		
Simulat* glasses*		
Gait* simulat*		
Tinnitus* simulat*		
Tremor* simulat*		
COPD* simulat*		
Knee* wrap*		
Overshoe*		
GERT*		
Age* suit*		
GERontologic*		
Weight* vest*		
Age* simulat*		
Agi* simulat*		
Ag* simulat*		
Simulat* suit*		
Age* suit*		
Simulat*-base* teach*		
Simulat*-base* training*		
Simulat*-base* educat*		
Simulat*-base* learn*		
Visual* impair* glasses*		
Diabet* simulat*		
"in patient's shoe*"		
Disast* simulat*		
Ostom* simulat*		
Wheelchair* simulat*		
Fat* suit*		

In keeping with our subjectivist epistemological position, we included a wide range of article types as legitimate sources of knowledge, including empirical-based research, commentaries and editorials that aligned with our research objectives. Our search resulted in a sample of 516 citations which were exported to Covidence Systematic Review Software© (Melbourne, Australia 2021) for review.

#### Stage 3: Article selection

From the initial list of 516 citations, 146 duplicates were removed leaving 370 articles. Two members of the research team (MK and GJG) independently screened all 370 abstracts, rejecting 227 articles that were deemed not within scope. Conflicts in selection between MK and GJG were discussed with JR until a consensus was achieved between the research team.

**Fig. 1:**
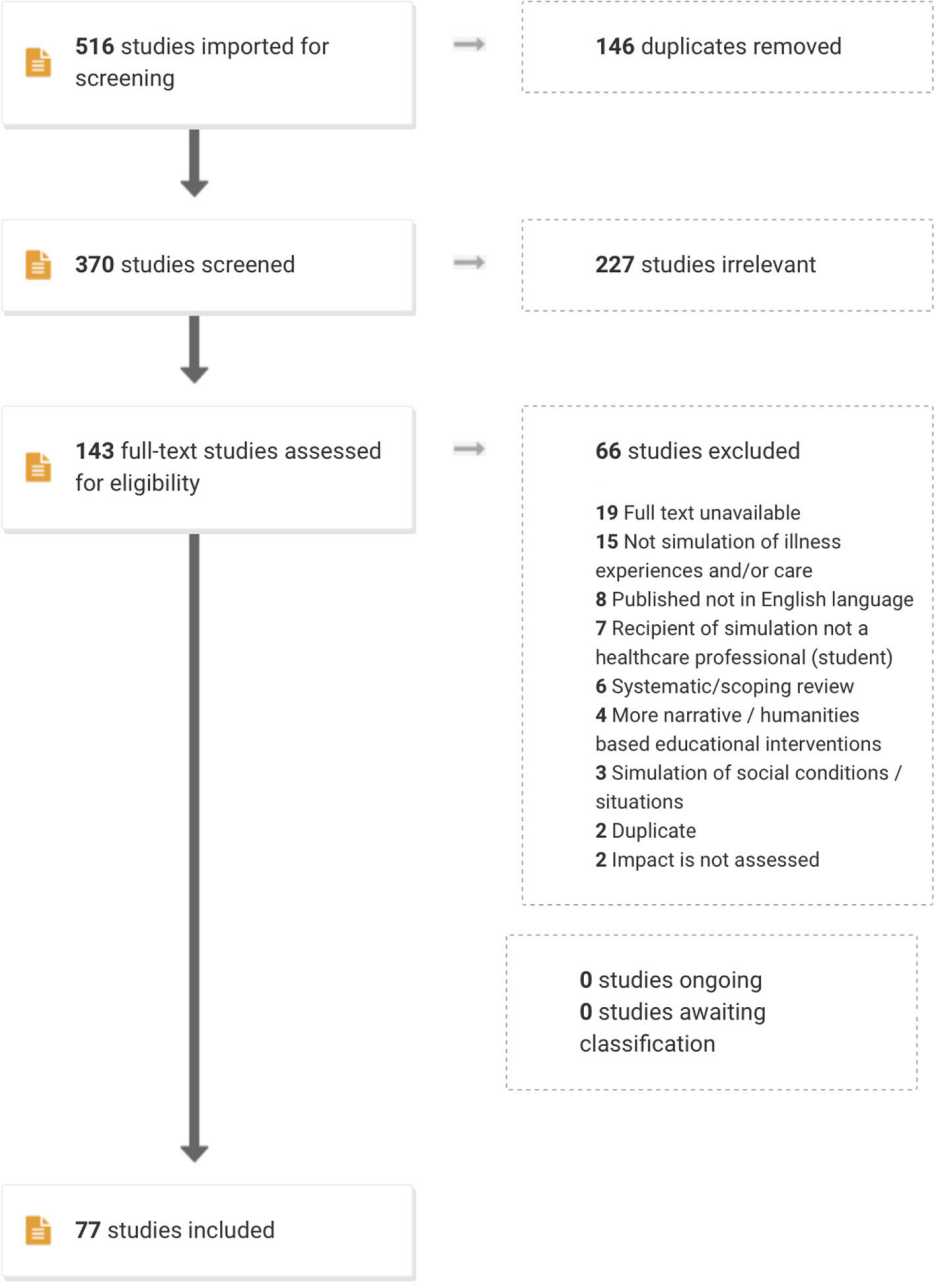
PRISMA flowchart of the screening and selection process

A priori inclusion/exclusion criteria were initially developed and refined iteratively throughout the selection process. Articles were included that pertained to (1) simulation-based methods of creating illness/treatment learning experiences and (2) HCP training (including students) that were (3) published in English language and for which (4) full text was available. Articles were excluded if they did not meet the inclusion criteria and if they (1) used computer-generated simulations of patient experiences (i.e. not embodied as physical simulations), (2) described more narrative/humanities-based educational interventions (i.e. we wanted to have a greater focus on experiential learning rather than, for example, creative writing or imagery about illness experiences), (3) involved simulation of mainly social conditions/situations (i.e. for example, poverty, quality of housing, homelessness, educational attainment), (4) were duplicates or systematic/scoping reviews or (5) did not measure impact. Full texts were recovered, and exclusion/inclusion criteria were applied once again. Disagreements between reviewers were settled through discussion until a consensus was achieved between the research team members. Of the remaining 143, the full-text articles were read and a further 66 were excluded. Additionally, articles’ reference lists were cross-checked to ensure no eligible studies were excluded. A total of 77 articles met our inclusion criteria. Figure 1 illustrates the PRISMA flowchart of the screening process.

#### Stage 4: Charting the data

After reading the selected full-text articles, all of the research team devised a data extraction template using Microsoft Excel (Microsoft, Redmond, USA) (see Additional file 1). The data template enabled capture of extracted data that was organised to address our research question. Key descriptive details of the article were extracted including author name(s), year of publication, journal, category of article type, location of research, methodological approach (if applicable) and the relevant HCP discipline.

Details of the simulated illness/treatment experience were also captured, including duration and type of simulation as well as the length of follow-up. Finally, information pertaining to the impact on health professions’ empathic skills was also captured, while documenting outcomes and their assessment with authors’ conclusions. MK charted the data from the selected articles. They were reviewed by the entire research team and any discrepancies were resolved by consensus.

#### Stage 5: Collating, summarising and reporting the results

Given the heterogeneity of the extracted data, a quantitative and qualitative approach was used to generate a map of the literature included in this review. A basic numerical analysis of the distribution and nature of the included articles was performed. Our main area of focus was to map what is known about the methods of creating simulated illness and treatment experiences. Given our subjectivist stance, we used a thematic analytical approach to report our findings thematically, informed by the charted data [49]. This was a collective and iterative process as we gained a growing understanding of the findings.

## Results

### Publication characteristics

Of the 77 articles selected for this scoping review, 52 (68%) originated from the USA, 11 (14%) from the UK, 4 from Australia, 3 Canada, 2 South Korea and 1 from each: New Zealand, Malaysia, Brazil, Taiwan and the Netherlands. Fifty-seven (74%) were published since the beginning of 2011 (i.e. 10 years before this review). The publications varied in their type of article category: 37 (48%) of studies were qualitative, 17 (22%) used a mixed-methods model, and a variety of other articles including descriptive (4 (5%)) and pilot pieces (2 (3%)) were analysed.

A wide range of HCP disciplines were represented as participants in the studies selected for this review. Overall, 61 (79%) studies included students (from which 18% were medical, 29% pharmacy, 42% nursing and 11% other healthcare students) and 16 (21%) involved HCPs.

### Illness and treatment experiences simulated

A diverse range of illness experiences were created for the purposes of HCPs’ training. Only 1 study, respectively, looked into the simulation of melanoma, psoriasis, inflammatory bowel disease, acquired brain injury, neuro-disability, urinary incontinence, palliative care, disaster drill and promotion of cultural awareness. The greatest number of studies (19) simulated old age and its associated symptoms (5% looked into sensory, auditory and visual impairment as well as deafness, and 8% researched dementia). Three combined and singular illness studies were conducted to explore the effects of physical disability. The second most common simulated condition was auditory hallucinations (15 (20%)), with separate studies exploring the point of view of patients with schizophrenia (2 (3%)). Twelve (16%) studies carried out diabetes simulations. Additionally, studies were conducted to simulate patienthood (2 (3%)), medication management (2 (3%)) and ostomy care (3 (4%)). Obesity simulations (3 (4%)) used bariatric suits to investigate struggles encountered by extremely overweight patients.

### Methods of simulating illness experiences

An array of methods and techniques were used to create simulated experiences of illness and treatment. Firstly, a number of studies described physical methods, i.e. glasses—scratched, yellow-tinted or welder’s goggles, to simulate blurred/tunnel vision, or normal yellowing of the lenses respectively, while others used The Simulation of Eye Disease Kit or 3D video to provide greater immersion for the learner. A number of studies contained more advanced equipment to simulate age-related macular degeneration (AMD) and glaucoma Sim-specs. Understandably, thick work gloves, simulating motor and sensory impairment, were used more often than The Tremor Kit. Ear cotton plugs and headphones (white noise/non-contingent distracting audio, emulating auditory loss) commonly appeared in simulations of deafness, presbycusis and especially auditory simulations associated with mental illnesses, such as schizophrenia.

Audio devices such as an mp3 player and headphones were the general equipment used in auditory experiments. Audio recording from ‘Hearing Voices That Are Distressing’ was commonly used for auditory hallucination studies. An alternative scenario of interaction (low-fidelity) while people talk behind the subject was additionally investigated. Interestingly, an effect of an immersive art exhibition ‘Altered States of Consciousness’ was evaluated—where subjects not only listened to, and experienced, auditory hallucinations but also observed art at the same time.

For disabilities, simulations most commonly used a manual wheelchair; nonetheless, crutches and walking canes were often mentioned especially while simulating old age or a mixture of conditions. To simulate muscle weakness in limbs, weights around chosen body areas (i.e. side of a body (stroke), wrist) were assumed as a most proximate experience equivalent to the addressed condition. Reduced joint mobility and arthritis were replicated with restrictors on elbows/knees, insoles with corn kernels and back protectors. The hemiparesis, age (physical limitation) and GERontological test suit were some of the suits used within simulation studies [37, 38, 52–54]. Fifty percent of studies simulating dementia used the Virtual Dementia Tour.

Visual aids, such as tattoos (melanoma, psoriasis), make-up (disaster drill, patienthood), non-invasive appliances (ostomy, incontinence undergarments, oral ‘medication’) and invasive procedures (‘insulin’ injections with saline) were frequently considered a good practice providing the highest chance of targeted embodiment.

Adherence to a diabetic diet and daily blood glucose logs were observed as having the highest success rates. Success rates referred to simulation being successful in helping participants increase their empathy levels, raising understanding of affected patients and finding effective management strategies. Additionally, it indicated an enhanced overall ability of purposefully ‘step into a person living with diabetes shoes’. A majority of studies tried to incorporate everyday tasks (i.e. going up/down the stairs, dressing/undressing, eating, pouring a drink, getting around their ‘living’ area; performing daily tasks such as shopping or using public transport) during their care/illness experience, providing a better point of view of the struggles encountered by a person with the condition.

### Impact of simulated experience on learners

A variety of approaches were used to determine the impact, if any, of the illness simulation experiences on learners. With respect to impact on learners, 27% deployed more quantitative methods (for example standardised questionnaires such as The Jefferson Scale of Physician Empathy), 49% utilised qualitative methods (i.e. thematic analysis of interviews) and 30% had a mixture of qualitative and quantitative approach. Impact was more often determined in the short term following the simulation experience, with some studies considering more long-term impact (20%). In the majority (87%) of articles selected for this review, simulation of illness experience had a positive impact on learners. However, in some instances (7%), there appeared to be no impact or more concerning—a negative impact on learners (6%). For example, auditory hallucination studies conducted by Brown et al. reported decreased willingness to help or interact with individuals with a mental illness, and they did not engender goodwill or a desire to have contact, but rather facilitated social distance and negative emotions, as well as an increase in attitudes regarding forced treatment. A sense of suspicion and less positive attitudes toward older adults was likewise observed in some simulations of old age.

A range of emotions could be evoked in learners by simulations of illness experiences. More often, these emotions could be more negative—including frustration, embarrassment and at times anger. For example, subjects’ frustration and impatience with peers for a lack of insight about the difficulties encountered when simulating old age, dealing with disabilities or facing difficulties completing tasks. Additionally, loss of independence throughout paralysis or impairment simulations left the majority of participants feeling vulnerable. Such emotions were more from the viewpoint of being the imagined patient and how they would be experiencing aspects of their illness or treatment. In most instances, such emotional responses were well tolerated by learners in the pursuit of their professional development. However, there were occurrences reported that additional measures were required to ensure learner wellbeing (e.g. an extended debrief) and psychological safety. For instance, in Addison and Morley simulation of palliative care, besides commonly shared ‘desperation, fear, anxiety, panic and exhaustion’, some subjects also displayed strong emotional responses to the activity and required additional post-experience debriefing to process their feelings.

Overwhelmingly, the simulation of illness experiences reported in the selected articles offered learners not just physical, but also at time cognitive, emotional and social dimension insights and awareness about illness experiences. For example, participants of diabetes simulations often acknowledged the challenge of needing privacy for insulin self-injection. Additionally, during simulations of chronic conditions, shift work, social occasions and family activities resulted in occasional but manageable difficulties (i.e. finding time for glucose monitoring at the recommended time periods and performing self-injection appeared to cause a significant amount of inconvenience when occurring in the middle of the shift or during a family celebration) [55, 56]. Studies also noted a link between the reality of living with obesity and experiences of wearing bariatric suits. Spatial awareness issues and environmental limitations enlightened participants that living with obesity may lead to severe social disengagement and isolation, as the effort required to carry out daily activities and mobilise was demotivating and monumental. Participants in various simulations shared an overwhelming feeling of awareness and practical understanding of the patient’s perspective going beyond the basic intellectual knowledge. Often such holistic simulation experiences would provide learners with a greater appreciation for what their patients may be experiencing. Aside from some of the main features of the condition, often, the ‘taken for granted’ impact that illness can have on individuals drew particular attention for learners. For example, simple daily tasks (for example undressing/dressing, moving around in the environment) became monumental, time-consuming and humbling when performed while wearing a GERontological/hemiparesis suit. Furthermore, the ability to make meaningful choices, both small and large (choice of clothing/meals; timing of the procedures), really counted when dependency was forced upon the subjects, providing an opportunity to redeem at least a part of lost dignity and integrity. More often, learners would internalise their experiences of illness and critically reflect on their role as healthcare providers.

While often the emotions that were experienced during the simulation were more negative, a transition to more positive, empowering emotions was often experienced by learners as they imagined their professional relationships with patients in the future. Often, learners gained a greater sense of empathy towards their patients. Not only an imagined empathy but also activating a desire to demonstrate empathic care towards their patients in the future. For example, providing more time, making sure conveyed information is well understood, maintaining eye contact, listening and providing reassurance were cited while interacting with patients who have dementia, or being more aware to refer to sensory services when patients present with sensory impairment, and explicitly asking people about their physical limitations (sight or hearing) to gain a better understanding of its impact on patients’ medication management. Additionally, a more uniform approach in management and a realistic acquaintance with the problems of compliance was adopted by subjects of diabetes simulations. In Skoy et al. study, 40% of participants noted positive gain of greater patience, understanding and appreciation toward what people that hear distressing voices encounter every day as well as an increase in awareness and enthusiasm for being a patient’s advocate. In many instances, the simulation of illness experiences generated a range of measures that they could translate into practice in order to demonstrate empathic care (i.e. adapting communication skills with patients).

## Discussion

For the first time, this scoping review provides an overview of methods used to simulate illness experiences for the benefit of HCP training. Our discoveries contribute to the growing field of literature focusing on person-centred care and empathy in the delivery of care [57]. A diverse range of methods and techniques exist that have been utilised by a wide range of HCP, and students, in their training. From relatively simple methods, such as wearing a transfer tattoo of a melanoma—to more sophisticated methods, such as wearing an ageing suit—these methods have been used to provide individuals with insights into illness and treatment experiences. In this way, they have attempted to provide a point-of-view perspective of the many dimensions of illness experiences—not just on a physical level, but also at times cognitively, emotionally and in the social dimension. It is worth noting that such simulations were not restricted to more medical conditions but included mental health conditions such as schizophrenia and dementia.

### Wide range of methods

Two general approaches to simulating illness experiences were apparent throughout the scope. The first involved the creation and transfer of conditioned/illness environment by proposing limitations, such as sight impairment simulation spectacles. The second part consisted of the incorporation of everyday tasks *while* being immersed in the simulation experience/equipment. A wide range of methods were observed throughout the studies indicating the emergence of increasing interest and resourcefulness for new ideas and techniques within the field. There was no unanimous approach to the restriction of movement, which may indicate insufficient simulation through the experiences studied, encouraging a continual search for a closer replication. The emergence of a dominant single piece of equipment, such as is seen with the use of headphones, indicates the success of this method. It is worth noting that the majority of diabetes simulations used a similar template while simulating the experience, but none of them examined a simulation of diabetic neuropathy, which could provide a deeper insight for HCPs of the emotions that diabetic patients encounter. Additional assumption of the role was often needed to facilitate a truly immersive experience. Encouraging students to perceive activities as a real diagnosis, affecting their lives from the point onwards, instead of seeing tasks just as a ‘temporary simulation’, enabled participants to receive feelings and emotions encountered by a newly diagnosed patient.

The second general approach included a performance of various tasks while wearing the equipment, which included walking around, going up/down the stairs, interacting with people, eating and reading/counting small items—tasks that seem effortless to the majority of the population who are in good health. Exposing participants in studies to similar, but at the same time different conditions, provided a stronger feeling of change. Feeling more ‘struck’ by a shift to a patient’s point of view, subjects were able to obtain deeper embodied (i.e. how we use our own bodily experience to understand our own emotional experience, and the experiences of others) perception and knowledge surpassing cognitive intelligence (the ‘Aha! moment’).

### The impact on empathic skills?

Generally, point-of-view simulation positively promoted attitudes towards empathic care and desires to be more understanding and patient and demonstrate kindness toward patients in general. These simulated experiences generally inspired intentions to deliver person-centred healthcare and equipped for better management of behavioural and psychological symptoms while sustaining ethical care practices especially to culturally and linguistically diverse groups. Overall, an increased confidence in teaching and performing self-management skills, recognition of the challenge and possibilities for improvement, feelings of empowerment and ownership and reduced negative perceptions and stigma were generated.

Some studies argued that simulating illnesses might be comprehended as a traumatic experience with following negative emotions. Distress and desire for greater social distance may undermine efforts to improve integration towards the patient cohort, even when subjects conveyed ‘understanding of what the disability experience is like’ and increased empathetic concern. The instance of auditory hallucinations conducted as a single experiential strategy was noted to possibly increase stigma in the participant. Outcomes such as an emerging sense of a patient’s absolute incompetence, or assigning ideas that they cannot successfully function in career and relationships mediums, were not congruent with the aims of experiential learning. Time of follow-up highlighted the need for prior/post-programme assessment accompanied by additional evaluation after internalisation of the experience, which would help to identify correspondence between attitudinal scores and the psychological measurements.

## Limitations

Despite the originality of the subject matter in this scoping review, it has to be considered within its limitations. Despite our robust search of the evidence base, it is likely that we may have omitted articles relevant to our research question. We intentionally excluded more humanities-based methods of sharing illness experience and appreciate that such methods could provide different and unique. Moreover, non-academic articles and grey literature were not included in this review, as they excluded impact evaluation on the subjects. It is possible that more data could be obtained by analysing research published in languages other than English. We did not conduct the optional stage 6 of Arksey’s and O’Malley’s scoping review methodology (i.e. consultation exercise). This stage is optional, and for pragmatic and resource reasons, this stage was not conducted.

## Recommendations

As a result of this scoping review, we have a number of recommendations. Firstly, having a repository of such point-of-view illness experience techniques would be of use to educators keen to integrate such methods in their training. Secondly, there is a need to consider the longer-term impact on empathic skills as a result of such point-of-view simulations. Moreover, it would be important to understand the potential negative impact on learners of such simulated learning experiences.

Thirdly, given the diverse range of approaches, development of an overarching pedagogical approach would help educators optimise their use in curricular and training programmes—for example, a systematic review could critically evaluate such approaches to simulating illness experiences and provide a guide to inform pedagogically practice. Lastly, we intentionally excluded experiences that were more social in focus (i.e. excluding simulations of poverty, quality of housing, homelessness, educational attainment), and these of course would be worth of further research.

## Conclusion

In conclusion, the findings of this scoping review provide an important insight into the extent and role of simulation in developing empathic skills in HCPs. A diverse range of methods and techniques exist that can afford a point-of-view perspective of aspects of living with illness or experiencing care. Beyond the physical feeling, many of these methods can evoke an emotional and embodied experience. A majority of these techniques appear to have a positive impact on empathy; however, the long-term impact remains largely unknown. Given the need to enhance empathic skills in HCPs, it could be argued that point-of-view simulation can offer a complementary approach in such training. Development of a guiding pedagogical framework, of how best to integrate such methods into training programmes and curricula, would help to optimise their use. As in the words of the American philosopher Henry Thoreau, ‘Could a greater miracle take place than for us to look through each other’s eyes for an instant’. While point-of-view simulations can never truly replicate illness experience, they have the potential to afford an embodied journey through some of the impacts and struggles encountered by people living with ill-health.

## Supplementary Information

**Additional file 1.** Link to the scoping review data extraction table (https://tinyurl.com/appendix1dataextractiontable).
